# Diagnosing autism spectrum disorder based on eye tracking technology using deep learning models

**DOI:** 10.3389/fmed.2025.1690177

**Published:** 2025-10-09

**Authors:** Mosleh Hmoud Al-Adhaileh, Saleh N. M. Alsubari, Abdullah H. Al-Nefaie, Sultan Ahmad, Asma Abdulmana Alhamadi

**Affiliations:** ^1^King Salman Center for Disability Research, Riyadh, Saudi Arabia; ^2^Deanship of E-Learning and Distance Education and Information Technology, King Faisal University, Al-Ahsa, Saudi Arabia; ^3^Department of Computer Science, College of Technology and Business, Riyadh Elem University, Riyadh, Saudi Arabia; ^4^Department of Quantitative Methods, School of Business, King Faisal University, Al-Ahsa, Saudi Arabia; ^5^Department of Computer Science, College of Computer Engineering and Sciences, Prince Sattam Bin Abdulaziz University, Al-Kharj, Saudi Arabia; ^6^School of Computer Science and Engineering, Lovely Professional University, Phagwara, India; ^7^Department of Humanities, College of Science and Theoretical Studies, Saudi Electronic University, Riyadh, Saudi Arabia

**Keywords:** autism spectrum disorder, eye-tracking, deep learning, diagnosing, ASD

## Abstract

**Introduction:**

Children with Autism Spectrum Disorder (ASD) often find it difficult to maintain eye contact, which is vital for social communication. Eye tracking (ET) technology helps determine how long children with ASD focus on someone, how frequently they do so, and in which direction their gaze moves. ET provides insights into social attention by enabling precise, real-time tracking of gaze patterns as individuals process social information visually. It is a dependable method for identifying and developing social attentional biomarkers, particularly in challenging conditions like ASD.

**Objective:**

This study aims to implement deep learning (DL) algorithms using eye-tracking data from social attention tasks involving children with ASD.

**Methods:**

The approach was tested using standard datasets collected from individuals with and without ASD through eye-tracking technology. Convolutional neural networks (CNNs) and long short-term memory (LSTM) models were used to analyze data from children with ASD. Data preprocessing techniques addressed missing data and converted categorical features into numerical values. Mutual information-based feature selection was employed to reduce the feature set by identifying the most relevant features, thereby improving system performance. These features were then analyzed using LSTM and CNN-LSTM models to evaluate their potential for diagnosing ASD.

**Results:**

The experimental results showed that the highest accuracy achieved was 99.78% with the CNN-LSTM model. Furthermore, the findings indicated that the proposed method outperformed previous studies.

**Conclusion:**

The system successfully diagnosed ASD using the ET dataset. This approach shows promise for clinical application, assisting healthcare professionals in diagnosing ASD more accurately through advanced artificial intelligence technology.

## 1 Introduction

Autism is a condition characterized by various signs that affect an individual’s communication, behavior, and social interaction. Autism has recently increased worldwide. The numerous symptoms displayed by autistic children make diagnosis more challenging (1–3). Autism is typically diagnosed by a team of professionals who observe the child’s behavior, a process that takes time and can be prone to errors, since the behavior of an autistic child may resemble that of children with other psychiatric conditions. Therefore, technical innovations are needed to develop alternative diagnostic methods (4).

Computer-aided (CA) systems are becoming increasingly important in diagnosing ASD (5). This category includes electroencephalography (EEG) (6), magnetic resonance imaging (MRI) (7), and ET (8). Genetic testing, eye-tracking (ET), facial features (9), emotion analysis (10), facial landmark identification (11), robot-assisted interaction, and eye contact training (12), as well as brain imaging, are all non-invasive methods to record, track, and measure eye movements or the specific point where a person’s eyes focus on a picture (3). These techniques help identify ASD in younger children, especially those who do not display obvious signs or symptoms, allowing for timely support and therapy. Understanding and interpreting ET scanpaths requires significant expertise in the field. Kids with ASD typically have difficulty focusing, which shows as unstable eye movements and reduced attention to important stimuli. Measuring eye movement fixation with scan path devices can be challenging because participants’ focal points may vary greatly depending on their interests and the experimental context. Traditional methods of diagnosing ASD are helpful, but they often rely on clinical interviews and behavioral observations, which some view as time-consuming, costly, and subjective. These approaches can also delay early diagnosis, particularly in younger children who may not yet show clear behavioral signs. When working with complex data streams, artificial intelligence (AI) techniques can significantly improve pattern recognition and prediction accuracy (13). Automation has the potential to simplify the identification of individuals with ASD by making the process more objective, faster, and precise.

The new AI model utilizes DL and machine learning (ML) techniques to improve existing methods by integrating objective biomarkers with clinical expertise (14, 15). These tools can replace traditional diagnostic approaches but are more effectively used as screening tools in support systems for healthcare providers. The aim is to increase accuracy and speed, leading to earlier and more reliable diagnoses. This approach seeks to accelerate the diagnosis process, reduce the time needed for diagnosis, and lessen the workload for healthcare workers (16–18).

### 1.1 Research question

- What is the appropriate feature selection method used to select the essential features?
- What is the approach of the DL model for diagnosing ASD bae on eye-tracking.
- How can DL assist in diagnosing ASD at early stages using eye-tracking technology?
- Developing an eye-tracking system based on the CNN-LSTM model for diagnosing ASD.
- Identifying the appropriate feature selection strategy to identify the significant features related to the ASD component that can assist the deep learning model in achieving high accuracy.
- The proposed approach achieves accuracy superior to prior studies by employing cross-validation methods.

This study aims to present an improved version of the autism recognition model by leveraging the benefits of deep learning through enhanced training and testing processes, including stratified cross-validation and feature selection methods. The main contribution is to refine the existing process that uses the same dataset for diagnosing ASD (19, 20). We are tracking the contributions of this research. The collection includes eye-tracking data gathered from 29 children diagnosed with ASD and 30 typically developing (TD) children. During data collection, the children remained engaged with both still images (such as balloons and cartoon characters) and moving videos. This study achieved an impressive accuracy level of 99.78% with the CNN-LSTM, marking a significant step forward in innovation.

## 2 Background

This section summarizes the studies used to analyze and diagnose ASD. Several studies and methods have been conducted for diagnosing ASD. Such methods include brain imaging, kinematic analysis, eye tracking, and more. Many research studies have employed DL and ML algorithms to confirm a diagnosis or assist in initial detection of ASD.

Zhao et al. (21) examined the effectiveness of eye-tracking data from in-person talks in reliably recognizing individuals with ASD. The researchers used four ML models: support vector machine (SVM), linear discriminant analysis, decision tree, and random forest. When variables related to ocular fixation and session time were included, the SVM model achieved a maximum testing accuracy of 92.31%. This outperformed methods that only used visual fixation characteristics or session length. Fadhel and Hussein (22) applied an ML approach to identify factors that hinder children’s growth and development. The system achieved 89% accuracy, detecting subtle differences that are difficult for the human eye to perceive. This study demonstrates that ML surpasses traditional methods in diagnosing autism because it is faster, more accurate, and more effective. Cilia et al. (23) note that early diagnosis of ASD is challenging due to cognitive testing, clinical exams, and symptom variability. This article proposes incorporating eye tracking into the ASD screening process. The study involved 59 school-aged volunteers who viewed age-appropriate images and videos related to social cognition. Eye-tracking scan paths were converted into visual representations of images, and a convolutional neural network was trained to categorize these images. The outcomes suggest that visualizations can streamline the diagnostic process and attain high accuracy. The approach may also apply to various conditions, especially neurodevelopmental disorders.

Kanhirakadavath et al. (24) conducted research using machine learning to assess the effectiveness of eye-tracking data in early autism screening for children. The deep neural network model outperformed existing models when tested on a benchmark dataset of 547 scan paths. This suggests that it could be utilized for quick and reliable autism screening, thereby enhancing physicians’ efficiency. Praveena et al. (25) employed Convolutional Neural Networks (CNN) to classify children with ASD and those with distinct developmental progress by analyzing fixation maps of the observer’s attention on the image. The model achieved 75.23% accuracy during testing, suggesting it could aid in analyzing visual input. Carette et al. (20) introduced ML methods to facilitate early detection of ASD in young children. This technology uses an ML approach to recognize eye-tracking patterns associated with ASD, converting scan paths into visual representations. The experimental results show that the predictions are quite accurate and simple to perform, with basic neural network models classifying data very effectively (AUC > 0.9). Elbattah et al. (26) developed an innovative method for diagnosing autism using AI tools. The research utilizes the Eye Gaze Fixes Map dataset and the ET Scanpath dataset to diagnose ASD. The hybrid model achieved higher accuracy rates of 96.1%.

Kang et al. (27) conducted research using ML to analyze EEG and eye-tracking data from toddlers, concentrating on their responses to face images of dissimilar competitors. Minimal redundancy was used for feature selection. The system showed that children with ASD paid more attention to hair and clothing than to facial features when observing face expressions. Current years have seen noteworthy advancements in research aimed at classifying and detecting ASD based on different ML techniques that utilize variables such as facial features and eye-tracking data (11, 28–32). Akter et al. (29) used an ML approach for detecting ASD by using ET technology. Satu et al. (33) applied multiple methodologies to identify ASD, aiming to determine the characteristics that distinguish autism from typical development across different ages. Erkan et al. (34) used RF algorithms to evaluate the effectiveness of each method in identifying ASD. Akter et al. (35) employed SVM to demonstrate higher performance on datasets involving toddlers, older children, and adults. Table 1 summarizes some notable research related to eye-tracking technologies for ASD identification. Carette et al. (20), applied simple neural networks with moderate accuracy (83%), while more advanced approaches like CNN-LSTM achieved superior performance, with Ahmed et al. (19) reporting 98.33% on clinical eye-tracking data. Other hybrid architectures, including CNN-LSTM (26) and CNN-GRU-ANN (36), also demonstrated robust accuracy above 84 and 93%, respectively. Deep neural networks (DNN) have been effective as well, with Kanhirakadavat et al. (24) reporting 93.28%, whereas MLP-based studies achieved around 87% (29, 30). In comparison, traditional machine learning methods such as SVM and logistic regression generally produced lower accuracies, ranging from 75 to 92% across face recognition, video, and gaze datasets (21, 25, 30, 37–39). Overall, these findings highlight the advantage of deep learning particularly hybrid architectures in capturing complex spatial and temporal patterns, leading to more reliable and accurate ASD diagnostic systems.

**Table 1:** Existing ASD systems.

Ref.	Type of dataset	Models	Purpose	Acc. %
Carette et al. (20)	Eye-tracking images	Neural network	Eye-tracking	83%
Ahmed et al. (19)	Eye-tracking clinical data	CNN-LSTM, LSTM	ASD diagnosing	98.33%
Akter et al. (29)	Eye-tracking Clinical data	MLP	Eye-tracking	87%
Liu et al. (37)	Face recognition	SVM	SD Diagnosing	85%
Zhao et al. (21)	Eye-tracking clinical data	SVM	ASD Diagnosing	92.31
Wan et al. (38)	Video ASD dataset	SVM	Detecting ASD	85
Akter et al. (30)	ASD face image	MLP	Classification of ASD based on MLP	87%
Alcañiz et al. (39)	Eye gaze data	SVM	ASD Diagnosing	91%
Elbattah et al. (26)	Eye-tracking clinical data	CNN and LSTM	ASD Diagnosing	84% CNN
Kanhirakadavat et al. (24)	Eye-tracking images	DNN	ASD Diagnosing	93.28%
Yaneva et al. (25)	Eye gaze data	Logistic regression	ASD diagnosing	75%
Aneva et al. (42)	Eye gaze image	CNN	ASD diagnosing	75.32%
Cilia et al. (36)	Eye-tracking clinical data	CNN-GRU-ANN		93.10

## 3 Materials and methods

**Figure 1:**
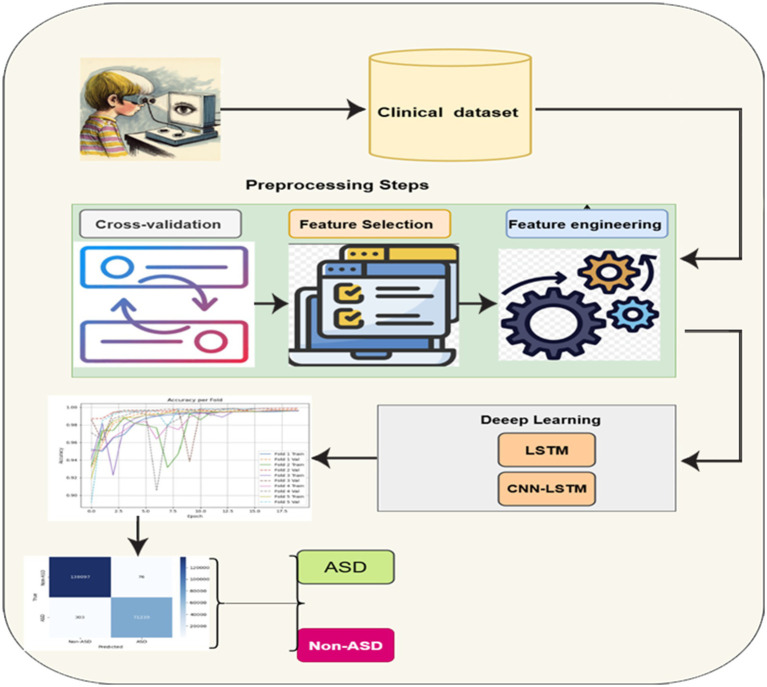
General framework of the proposed methodology.

This module presents the proposed method for developing a DL system to detect autism using eye-tracking data. The process starts with collecting raw gaze and behavioral features from participants as they view visual stimuli. These features are then processed and selected based on their relevance using mutual information scores. The most informative signals, such as tracking ratio, gaze coordinates, and CARS scores, are retained for modeling. The integrated DL model, which includes convolutional and recurrent layers, is trained to recognize subtle patterns in the selected features. This model captures both spatial and temporal aspects of gaze activity, enabling it to distinguish more accurately between autism and non-autism profiles. Cross-validation and early stopping are employed to ensure reliability and prevent overfitting during training. Figure 1 illustrates the framework of the proposed methodology. The details of the framework are discussed below.

### 3.1 Clinical dataset

This research work uses a benchmark and standard dataset (36) entitled “ET Dataset to Support the Research on ASD.” The dataset contains raw statistical information derived from eye-tracking experiments. It includes data collected from a total of 59 children, 29 diagnosed with ASD and 30 typically developing (TD) children, as summarized in Table 2. The dataset contains 1,048,575 instances.

**Table 2:** Summary participant group details.

#Gender	ASD group	Non-ASD group
#Female_numbers	4	17
#Male_numbers	25	13
#Total	29	30
#Total instance	1,048,575	
Age/mean	7.88	

The dataset used in this study was collected with a RED mobile ET operating at 60 Hz. The tracker was connected to a 17-inch display showing the visual stimuli during the experiment. Data collectors followed a controlled process in a dedicated experimental setting. Participants sat about 60 centimeters from the screen, enabling the eye tracker to record their gaze by detecting infrared reflections.

To effectively engage participants, the researchers used a mix of active and static visual stimuli. The dynamic content included short videos with engaging elements like cartoon characters and balloons, specifically created to attract children’s attention. The static stimuli comprised images of faces, objects, and scenes aimed at encouraging visual engagement. Each session lasted about five minutes, with the sequence and arrangement of items changing throughout the experiment. A key part of the stimulus design involved video clips of a human presenter delivering spoken content, intended to direct participant attention to specific elements on the screen, even if they were not visible all the time.

**Figure 2:**
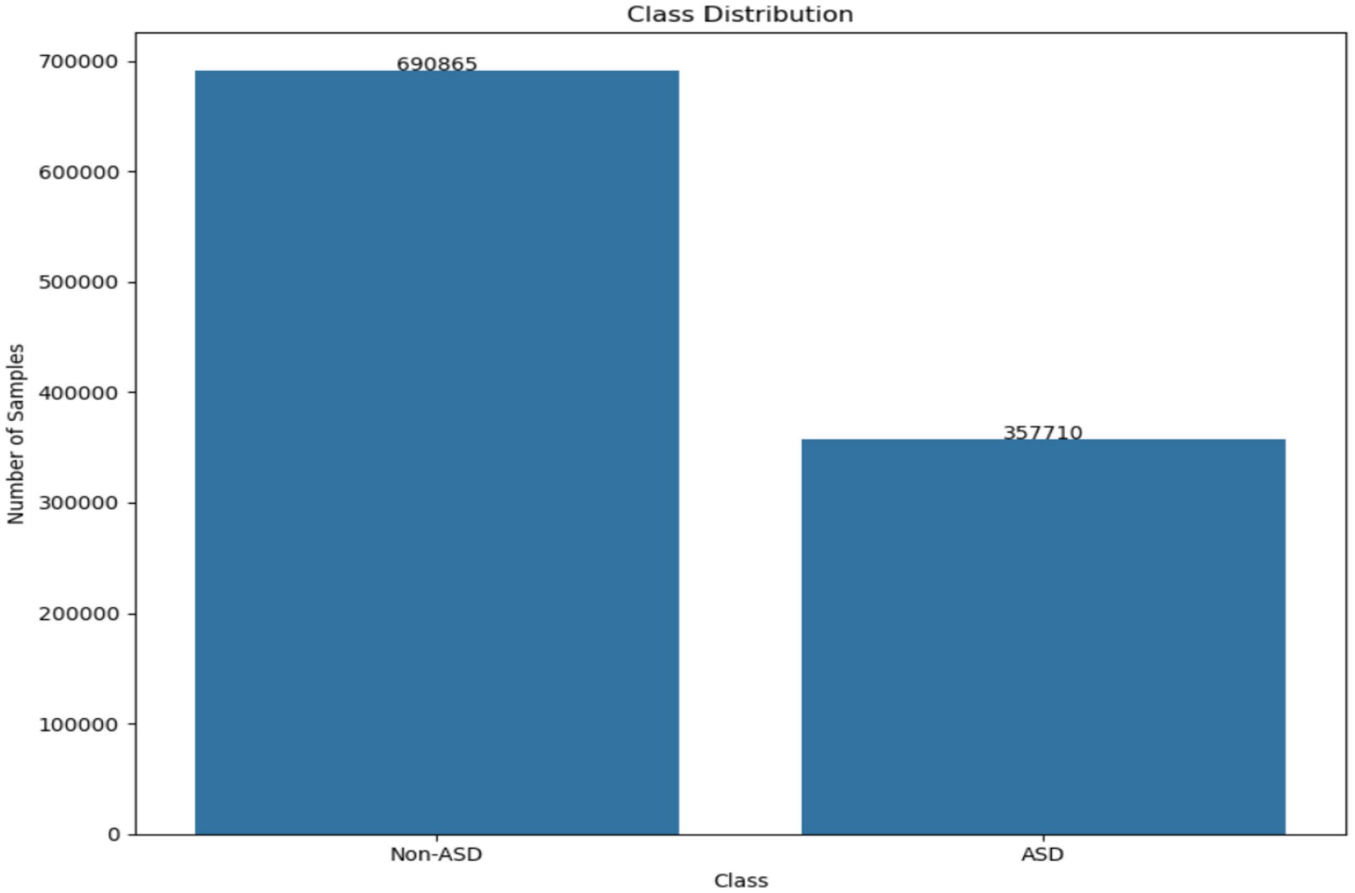
The class distribution existing in the dataset.

**Figure 3:**
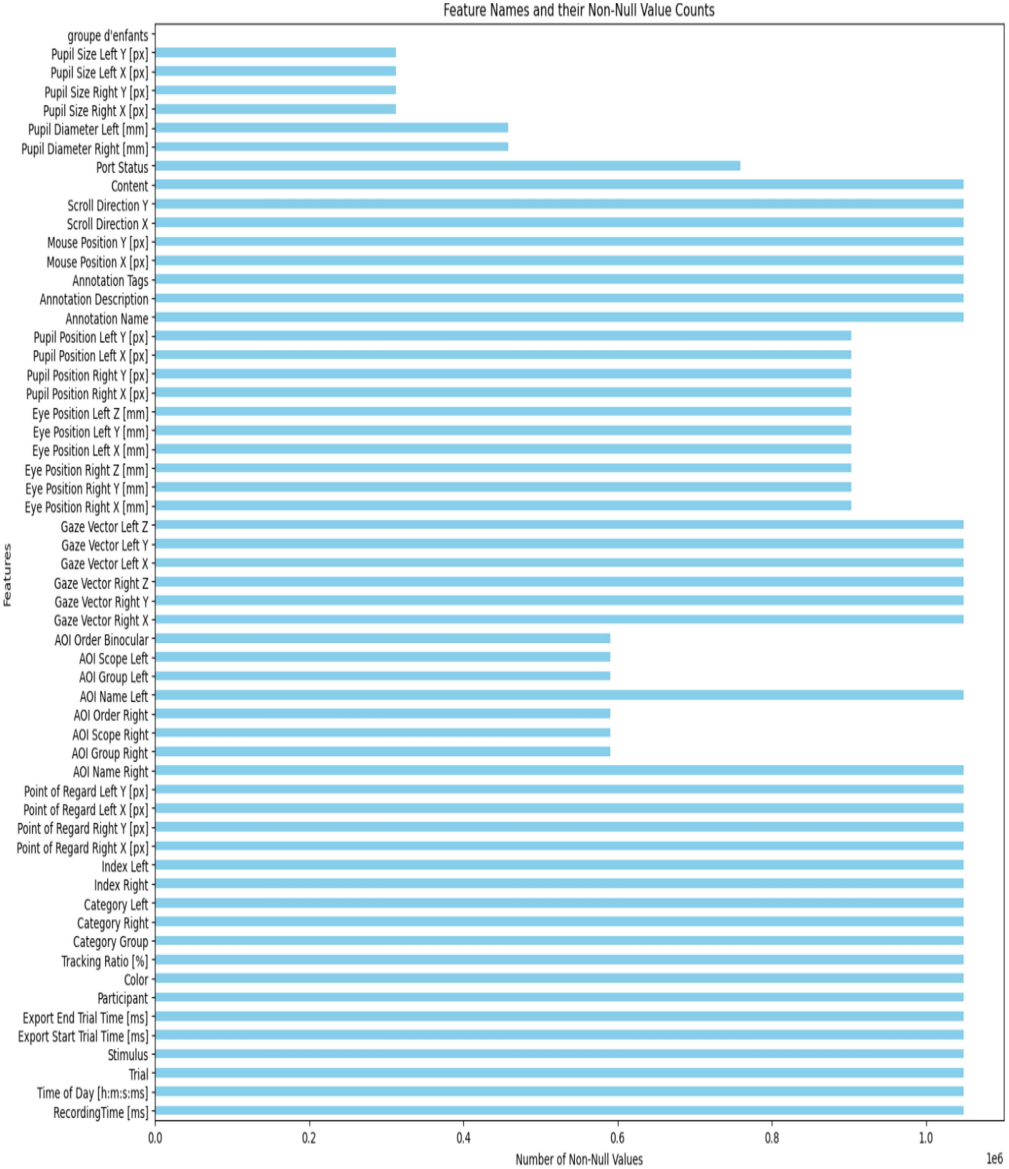
The number of non-values for each feature.

This setup enabled the collection of valuable data on eye contact, attention span, and engagement levels. The dataset offers insights into visual behavior through metrics like fixation patterns, saccadic movements, and blink rates. It helps distinguish the visual attention profiles of kids with ASD from those without it. The recorded dataset contains approximately 2.17 million rows of gaze statistics. Figure 2 illustrates how the classes are distributed within the dataset. Figure 3 displays a list of dataset features.

### 3.2 Data preprocessing

**Figure 4:**
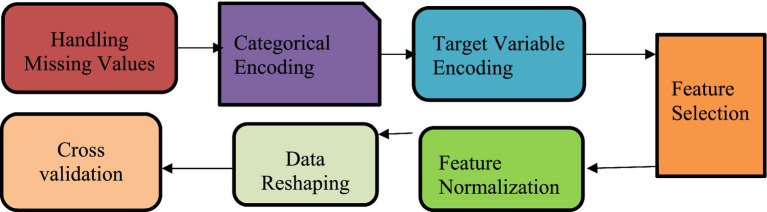
Preprocessing steps.

Preparing data is a vital step in any data analysis process. It involves organizing, cleaning, and transforming raw inputs to ensure the dataset is accurate and dependable. The goal at this stage is to make the data suitable for modeling by selecting the most relevant features, handling missing or incorrect values, and applying proper scaling. These steps help build a strong foundation for any analysis or machine learning tasks that follow. Figure 4 shows the data preprocessing steps performed.

#### 3.2.1 Handling missing values

**Figure 5:**
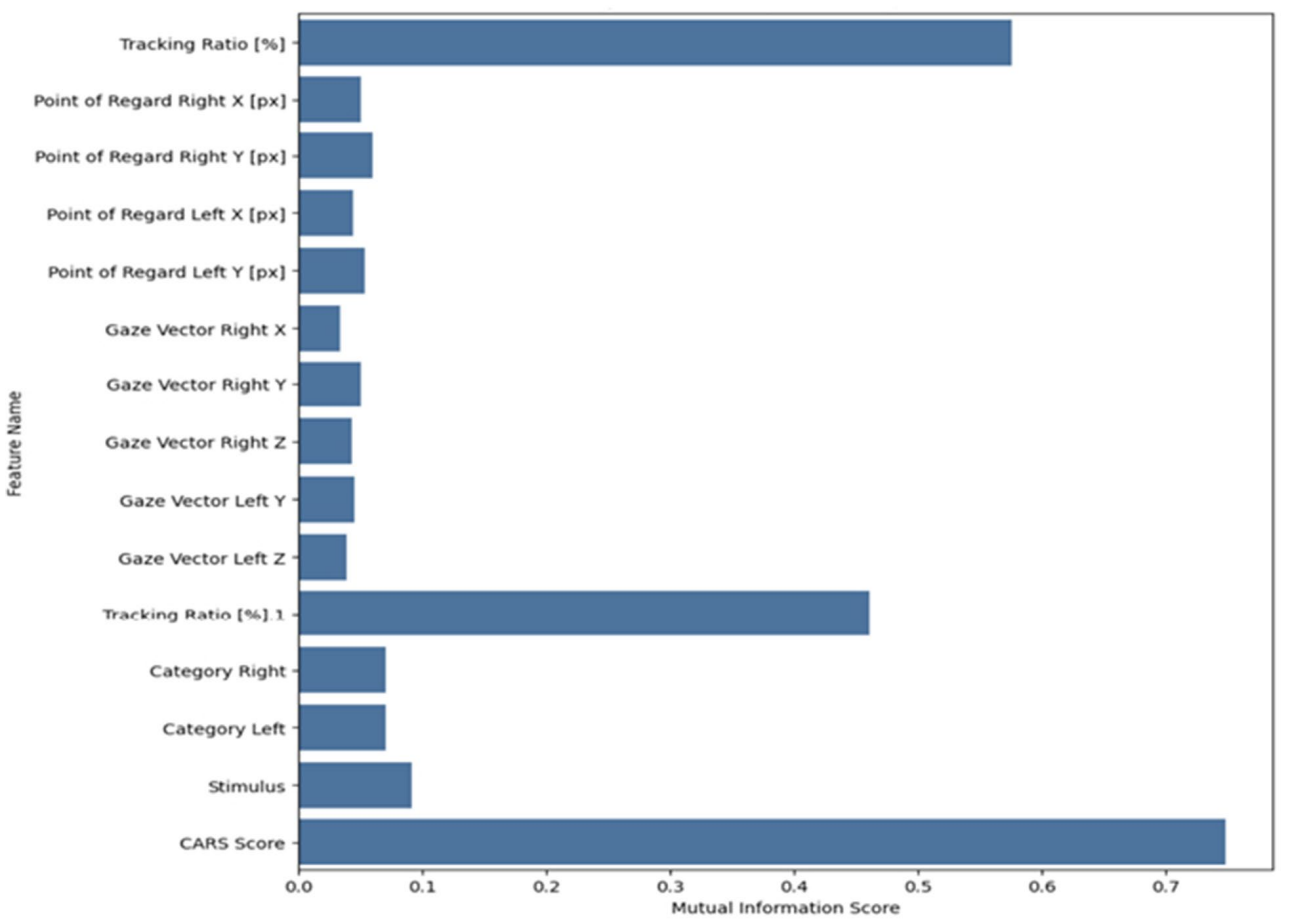
The importance of features using the mutual information approach.

The dataset had missing entries in several numerical features. To fix this while keeping the dataset’s structure and size, missing values were filled with the mean of each relevant feature. This method maintains the data’s central tendency and prevents losing informative instances that could happen with deletion. The amount of missing data for each feature is shown graphically in Figure 5.

#### 3.2.2 Categorical encoding

When working with the eye-tracking dataset, certain categorical features needed to be converted into a numerical format to ensure compatibility with the DL model. To do this, label encoding was used. This method assigns unique numeric values to each group in a variable, enabling the model to interpret and process the data effectively. Characteristics like (Trial, Stimulus, Color, Category Right, and Category Left) were among those transformed. This step is vital because most ML and DL approaches work with numerical input rather than categorical text.

#### 3.2.3 Target variable encoding

The target variable, which indicated participant group classification, was converted into binary form to support binary classification. This change was necessary to make class labels compatible with the model’s output layer and loss function.

#### 3.2.4 Feature selection

To reduce dimensionality and enhance the model’s performance, a mutual information-based feature selection method was employed to focus on the most relevant input features. The top 15 features with the strongest mutual relationship to the target variable were selected. This step reduced noise, improved computational efficiency, and minimized overfitting. Table 3 displays the selected features and their definitions.

**Table 3:** Selected features and definitions.

**NO.**	**Feature Name**
1	Tracking_Ratio [%]
2	Point_Regard_Right X [px]
3	Point_Regard_Right Y [px]
4	Point_Regard_Left X [px]
5	Point_Regard _Left Y [px]
6	Gaze_Vector_Right X
7	Gaze_Vector_Right Y
8	Gaze_Vector_Right Z
9	Gaze_Vector_Left X
10	Gaze_Vector_Left Y
11	Gaze_Vector_Left Z
12	Tracking_Ratio [%].1
13	Category_Right
14	Category_Left
15	#Stimulus
16	CARS_Score

**Figure 6:**
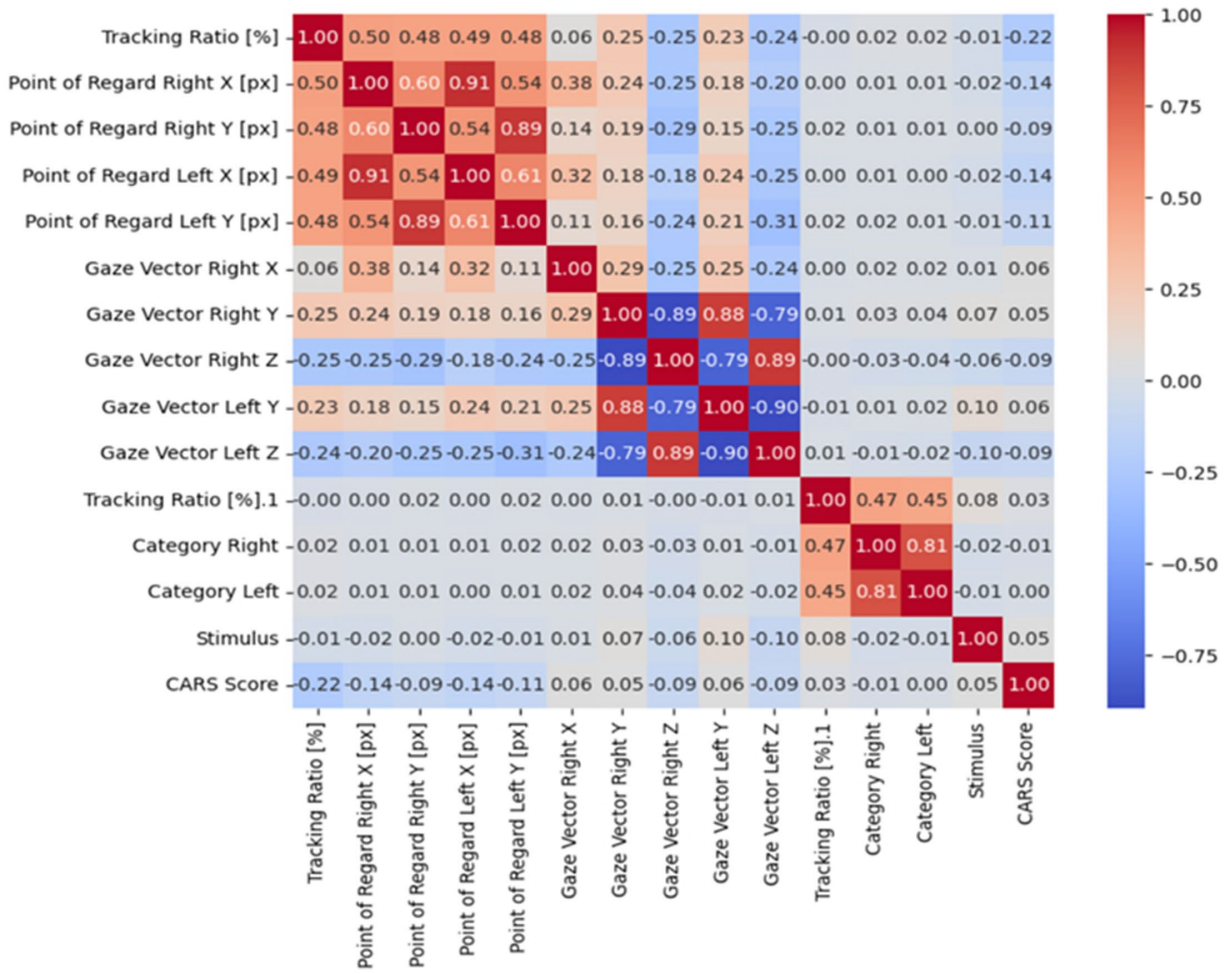
The correlation coefficient.

Mutual information was used as feature selection to assess the importance of each feature in relation to the target class. This approach measures the shared information between features and class labels, aiding in identifying which attributes are most helpful in differentiating between ASD and TD groups. By ranking features according to their mutual information scores, the model emphasizes those that most contribute to accurate classification. Figure 6 illustrates the significance of a set of features using the mutual information method.

The correlation between features can be effectively calculated using a correlation coefficient, which shows the strength and direction of their relationship (40). To visualize these correlations, a heatmap is often used because it provides an intuitive visual way to see how different features are related. By using a gradient color scale, the heatmap emphasizes areas of strong positive or negative relationships; darker shades typically indicate stronger positive correlations, while lighter or contrasting shades suggest negative or weaker links (41, 43). As shown in Figure 6, this visual approach makes it easier to understand complex interactions within the dataset, offering clearer insights into the patterns and dependencies among various features.

#### 3.2.5 Feature normalization

After feature selection, the remaining features were normalized using min–max scaling. This approach scaled the feature values to a consistent range between 0 and 1, which is crucial for optimizing convergence and ensuring stable gradient behavior during model training.

#### 3.2.6 Data reshaping

To match the input structure needed by the recurrent neural network, the feature matrix was reshaped into a three-dimensional format. Each sample was represented as a single timestep with multiple input features, enabling the use of sequential modeling techniques.

#### 3.2.7 Cross-validation strategy

**Figure 7:**
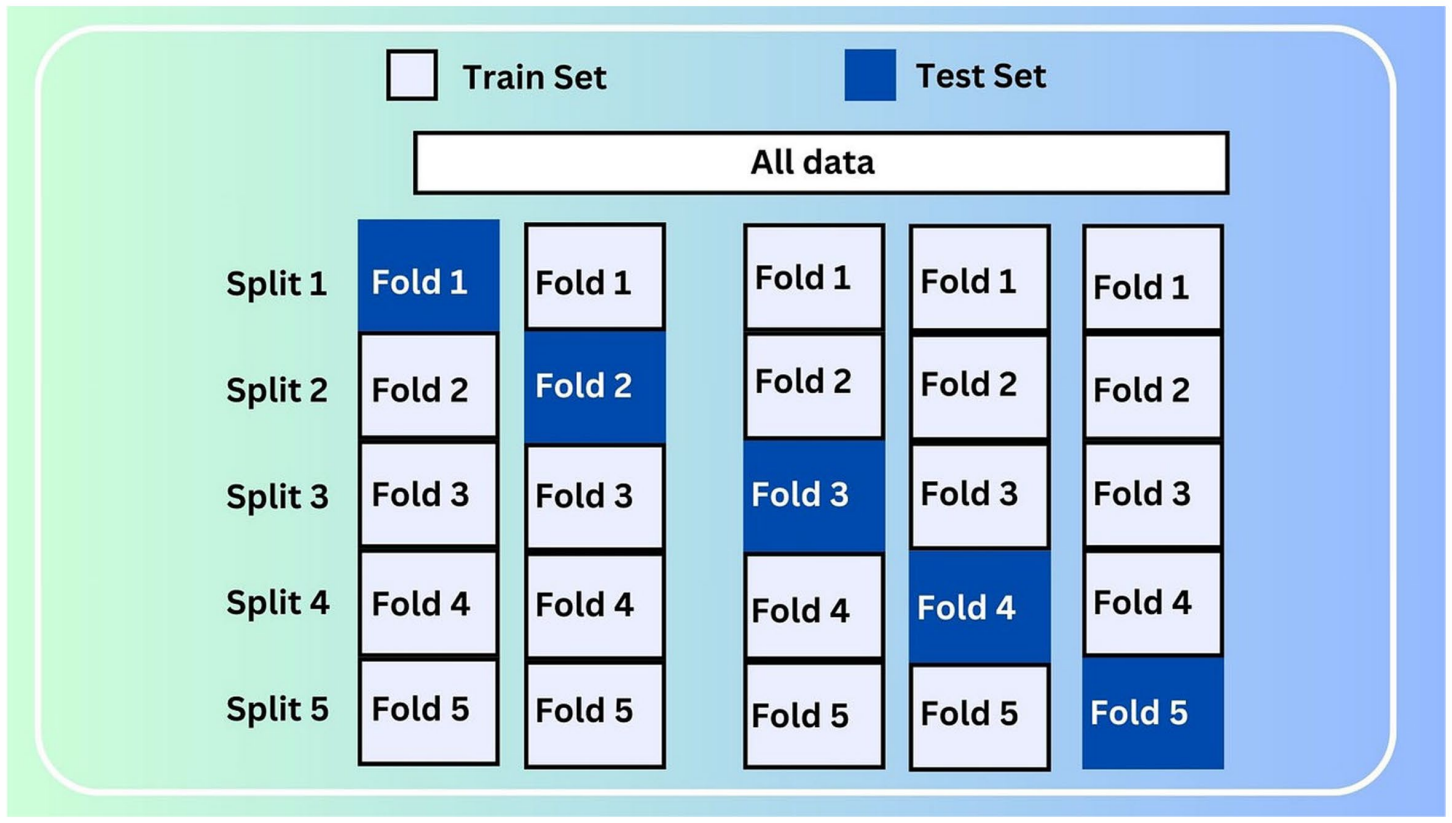
Stratified 5-fold cross-validation.

To ensure the strength and generalizability of the experimental outcomes, a stratified k-fold cross-validation technique with five splits was used. This method maintained the distribution of the target variable in each fold and allowed for evaluation across multiple data partitions. Figure 7 shows a graphical representation of the stratified 5-fold cross-validation strategy used.

### 3.3 Deep learning models

#### 3.3.1 LSTM model

The classification model in this study was built around a deep neural network that included a recurrent structure, specifically a LSTM layer. Even though the input consisted of only a single timestep, the model was designed to learn subtle patterns and interactions within the selected features. The core of the network was an LSTM layer with 64 memory units, serving as the main feature extractor. To prevent overfitting, dropout regularization was applied immediately afterward, deactivating 40% of the units randomly during training.

**Figure 8:**
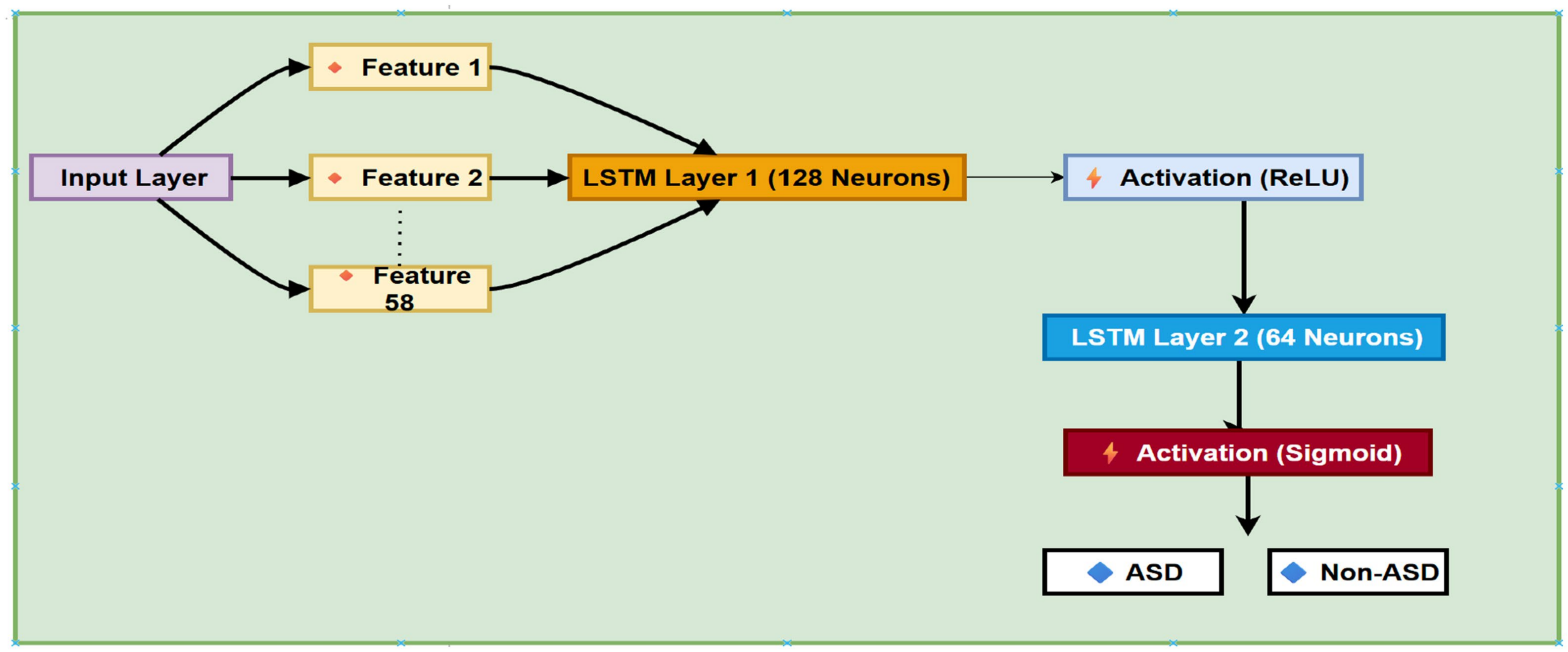
The architecture of LSTM model.

Following the LSTM, the network structure includes 32 neurons in a dense layer, along with a ReLU nonlinear activation function that enables the model to learn more abstract, higher-level representations of the training and validation data. The LSTM model architecture is shown in Figure 8.

Another dropout layer was added here as well, further decreasing the chance of overfitting. The final prediction used only one output neuron with a nonlinear sigmoid activation, giving a probability score for binary class sorting.

Furthermore, memory cells are considered the main components of the LSTM; each cell has three essential components called gates: the forget gate, input gate, and output gate. These gates are organized by sigmoid activation functions that determine how information flows through the cell. Specifically, the input gate decides which new data should be kept, the forget gate determines what information or data should be discarded from the cell’s memory, and the output gate decides what information is passed to the next step. These operations are guided by a set of equations that describe how data is processed within each cell. Equations (1–9) of LSTM model are as follows:


(1) $$f_{t}=\sigma (W_{f}.X_{t}+W_{f}.h_{t-1}+b_{f})$$

(2) $$i_{t}=\sigma (W_{c}.X_{t}+W_{i}.h_{t-1}+b_{i})$$

(3) $$C_{t}=(W_{f}*(.h_{t-1},x_{t})b_{f})$$

(4) $$o_{t}=\sigma (W_{o}+X_{t}+W_{o}.h_{t-1}+V_{o}.C_{t}+b_{o})$$

(5) $$h_{t}=o_{t}+\tanh(C_{t})$$

The input, hidden state, memory cell state, biases, and weights of the network are denoted by $W_{s}U_{s}b$ , while the input, forget, and output gates are represented t , $i_{-}t,f_{-}t,o_{-}t$ Respectively. The hyperbolic tangent activation function is represented by tanh, whereas the sigma function represents the nonlinear sigmoid activation function.

During the training process, the model was optimized and trained using binary cross-entropy loss, a common choice for two-class problems, especially when there is class imbalance. An adaptive optimization algorithm was employed to dynamically adjust the learning process, helping the model converge efficiently. To prevent overtraining, early stopping was implemented, stopping training if the validation loss did not improve after several epochs, with the best model weights automatically restored. The model’s evaluation performance was conducted using multiple cross-validation folds to verify the reliability and consistency of the results. Table 4 summarizes the LSTM model parameters used in its structure.

**Table 4:** The parameters used in the LSTM model structure.

Parameter	Value/description
Input shape	(1, k) – one timestep, k selected features
LSTM units	64 memory units
Dropout rate (after LSTM)	0.4
Dense layer units	32 neurons
Dense layer activation	ReLU (Rectified Linear Unit)
Dropout rate (after dense)	0.4
Output layer	1 neuron (sigmoid activation)
Optimizer	Adaptive optimizer (Adam) with learning rate = 0.001
Batch size	32
Number of epochs	Up to 20
Early stopping	Patience = 5 epochs, monitor = validation loss

#### 3.3.2 CNN-LSTM model

To support the binary classification of ASD using eye-tracking data, a DL model combining convolutional and recurrent layers was developed. The model’s input is a fixed-length feature vector created from preprocessed gaze data, capturing behavioral and statistical features relevant to ASD diagnosis.

**Figure 9:**
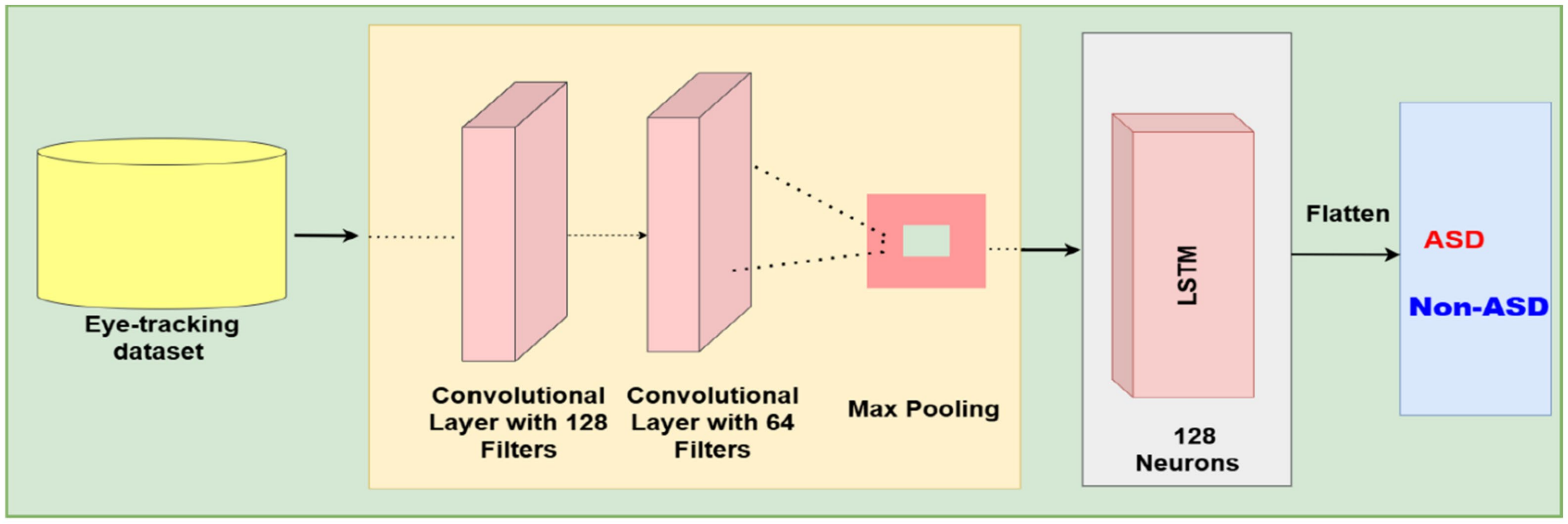
The structure of the CNN-LSTM based model.

Initially, local feature patterns were extracted using a one-dimensional convolutional layer, which helps capture spatial dependencies within the input vector. A pooling operation then followed to reduce noise and stabilize the extracted feature maps. The output was subsequently passed to a recurrent layer composed of LSTM units, allowing the model to recognize and capture temporal dependencies and sequential behavior in gaze data features, which are often indicative of atypical visual processing in ASD. Figure 9 shows the architecture of the CNN-LSTM model.

Subsequently, a fully connected layer was added to improve the learned representations, followed by dropout layers placed at key points in the architecture to prevent overfitting. The final classification was made using a single sigmoid-activated output neuron, providing a probabilistic prediction of class membership (ASD vs. non-ASD).

Binary cross-entropy served as the loss function for training the model, optimized with an adaptive gradient-based algorithm. Additionally, early stopping was employed based on validation loss with a fixed patience to ensure convergence and minimize overfitting. Training was performed in mini-batches to improve stability and computational efficiency. Table 5 outlines the parameters used in the CNN-LSTM architecture.

**Table 5:** Summary of the parameters used in the CNN-LSTM model architecture.

Component	Parameter/setting
Input shape	(1, k) — one timestep, k selected features
Convolutional layer	64 filters, kernel size = 3, activation = ReLU
Padding	Same (to preserve input dimensions)
Pooling layer	Max pooling, pool size = 1
LSTM layer	64 memory units
Dropout (after LSTM)	0.4
Dense layer	32 neurons, activation = ReLU
Dropout (after dense)	0.4
Output layer	1 neuron, activation = Sigmoid
Loss function	Binary Cross-Entropy
Optimizer	Adam, learning rate = 0.001
Batch size	32
Epochs	Up to 20
Early stopping	Patience = 5 epochs, monitor = validation loss

## 4 Experimental results

In this module, the experimental design of our study, containing the measurement metrics used and a summary of the DL model performance results, is presented.

### 4.1 Environment setup

The procedures in our experiments were performed on a laptop with a 7th-generation Intel Core i7 processor, 8 GB of RAM, and a GPU with 16 GB of dedicated memory. The deep learning models were built and tested using a suitable computational framework for neural network training. These software and hardware setups provided sufficient resources for practical model training and performance assessment.

### 4.2 Data splitting

The clinical autism dataset was split using a stratified 5-fold cross-validation method to ensure each fold had a balanced mix of ASD and non-ASD samples. This method enhances the robustness of model evaluation by reducing bias and variance, enabling the model to be trained and tested on representative data subsets.

### 4.3 Evaluation metrics

This subsection presents the evaluation of the performance of the proposed deep learning models used for autism detection in our experiments. A variety of evaluation and performance metrics, including sensitivity, precision, recall, accuracy, F1 score, and the confusion matrix, are employed for this purpose. Each of these measures offers a different perspective, highlighting various aspects of the model’s effectiveness and identifying areas where it performs well or may need improvement.


(6) $$\text{Accuracy}=\frac{\mathrm{TN}+\mathrm{TP}}{\mathrm{TP}+\mathrm{TN}+\mathrm{FN}+\mathrm{FP}}\times 100$$

(7) $$F1-\text{score}=2\times \frac{\;\text{Precision}\times \text{Recall}\;}{\;\text{Precision}+\text{Recall}\;}\times 100$$

(8) $$\text{Sensitivity}=\frac{\;\mathrm{TP}}{\;\mathrm{TP}+\mathrm{FP}\;}$$

(9) $$\text{Specificity}=\frac{\;\mathrm{TN}}{\;\mathrm{TN}+\mathrm{FN}}$$

### 4.4 Validation results of the LSTM model

The outcomes of testing the LSTM model for autism detection are presented in the subsection and shown in Table 6, which demonstrates that it performs consistently well across all five folds for both ASD and Non-ASD classifications. In nearly all cases, precision, recall, and F1-scores stay above 99%, indicating that the LSTM provides good performance at correctly classifying both positive (ASD) and negative (non-ASD) cases with very few mistakes. Non-ASD predictions have very balanced precision and recall, often approaching perfect agreement. At the same time, ASD predictions also reach similarly high values, showing that the model can capture subtle temporal patterns in eye-tracking data. The slight differences between folds suggest that the model’s generalization results are steady, with no significant decline in predictive quality across different data partitions. Overall, the LSTM model’s metrics confirm that it is strong and reliable at distinguishing between ASD and non-ASD participants.

**Table 6:** Summary of testing classification results of the LSTM model.

Fold No.	Labels	Accuracy	Precision	Recall	F1-score	Support
Fold 1	Non-ASD	99.67	99.87	99.63	99.63	138,173
	ASD		99.29	99.74	99.52	71,542
Fold 2	Non-ASD	99.89	99.85	99.98	99.91	
	ASD		99.97	99.70	99.83	
Fold 3	Non-ASD	99.50	99.33	99.92	99.62	
	ASD		99.85	99.70	99.27	
Fold 4	Non-ASD	99.69	99.75	99.78	99.77	
	ASD		99.57	99.52	99.55	
Fold 5	Non-ASD	99.76	99.64	100	99.82	
	ASD		100	99.30	99.65	
Average	Non-ASD	99.70	99.69	99.86	99.77	
	ASD		99.74	99.39	99.56	

**Figure 10:**
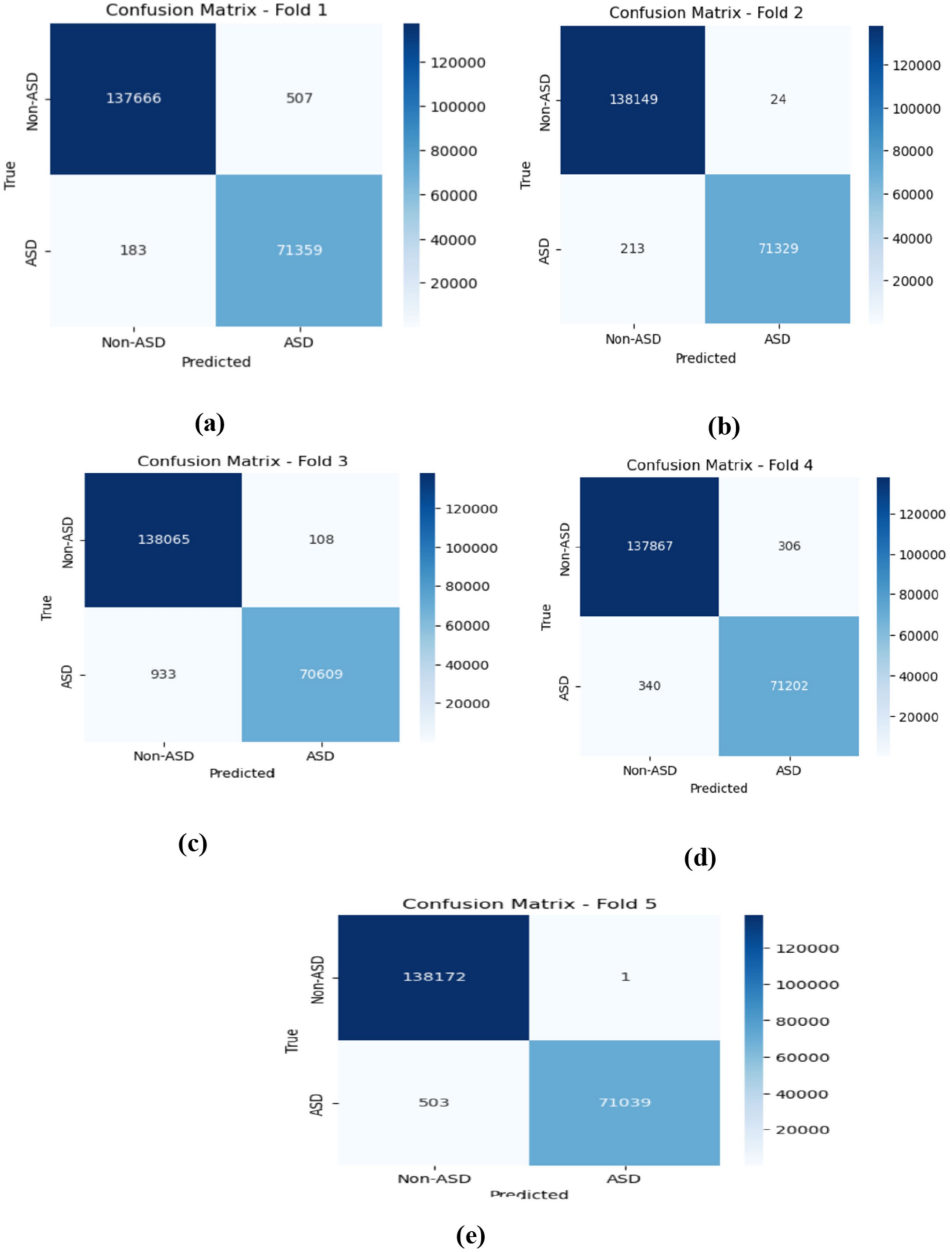
Displaying the confusion matrices obtained by the LSTM model within five-fold cross-validation.

On average, the LSTM model achieved excellent performance, with Non-ASD classifications showing 99.70% accuracy, 99.69% precision, 99.86% recall, and a 99.77% F1-score. For ASD classifications, the model demonstrated similarly strong results, with 99.74% accuracy, 99.39% precision, 99.56% recall, and a well-balanced F1-score. These averages highlight the model’s consistent ability to correctly classify both categories with minimal performance trade-offs, indicating high reliability and generalization across all folds. As shown in Figure 10, which represents the confusion matrices for the LSTM model, in Fold 1, the model demonstrated commendable performance, achieving 137,666 TN and 71,359 TP. The presence of only 507 and 183 FN underscores its effectiveness in accurately classifying instances. In Fold 2, the model maintained this strong trajectory, with 138,149 TN, a mere 24 FP, and 213 FN, reflecting high specificity and sensitivity in its predictions. Moving to Fold 3, the results revealed 138,065 TN and 70,609 TP, though the FN count increased to 933, indicating a slight rise in misclassifications while still demonstrating robust classification capabilities. In Fold 4, the model recorded 137,867 TN and 71,202 TP, accompanied by 306 FP and 340 FN, which points to a balanced performance across classifications. Finally, Fold 5 showcased the model’s impressive predictive accuracy with 138,172 TN and only 1 FP; however, the FN rose to 503, suggesting the necessity for further examination of these misclassifications to enhance overall performance. Figure 10 describes the model training and validation accuracies within five folds.

The accuracy curves for the LSTM model across the five folds offer a clear view of its training and validation performance throughout the epochs. In Fold 1, the training accuracy begins at around 0.96 and steadily rises to about 0.99 by the end, while the validation accuracy starts similarly at 0.96 and stays at a comparable level. This shows a good fit, with no significant overfitting. Moving to Fold 2, both training and validation accuracies follow a similar pattern, starting near 0.95 and approaching 1.00, indicating strong generalization of the model.

**Figure 11:**
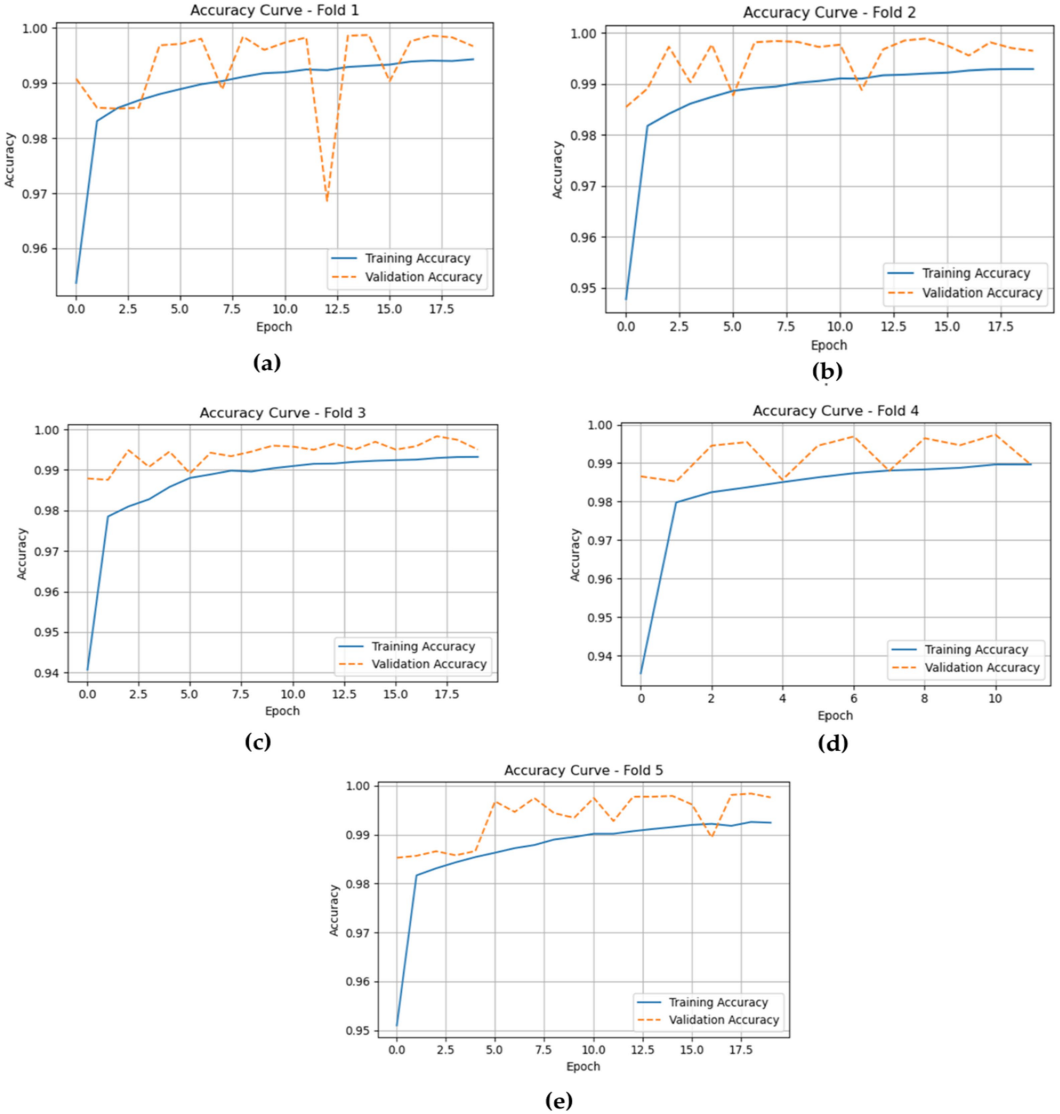
Displaying the model training and validation accuracies within five folds of the LSTM model. **(a)** fold 1 **(b)** fold 2 **(c)** fold 3 **(d)** fold 4 **(e)** fold 5.

In Fold 3, the training accuracy begins at approximately 0.94 and increases to nearly 1.00, with validation accuracy showing a similar upward trend and remaining high throughout. For Fold 4, training accuracy starts around 0.94 and gradually rises, stabilizing near 0.99, while validation accuracy reflects this improvement, indicating the model’s robustness across epochs. Finally, in Fold 5, training accuracy begins at roughly 0.95 and reaches about 0.99, with validation accuracy consistently close to this level, demonstrating strong performance without notable differences between training and validation results. Figure 11 shows the training and validation losses across five folds.

**Figure 12:**
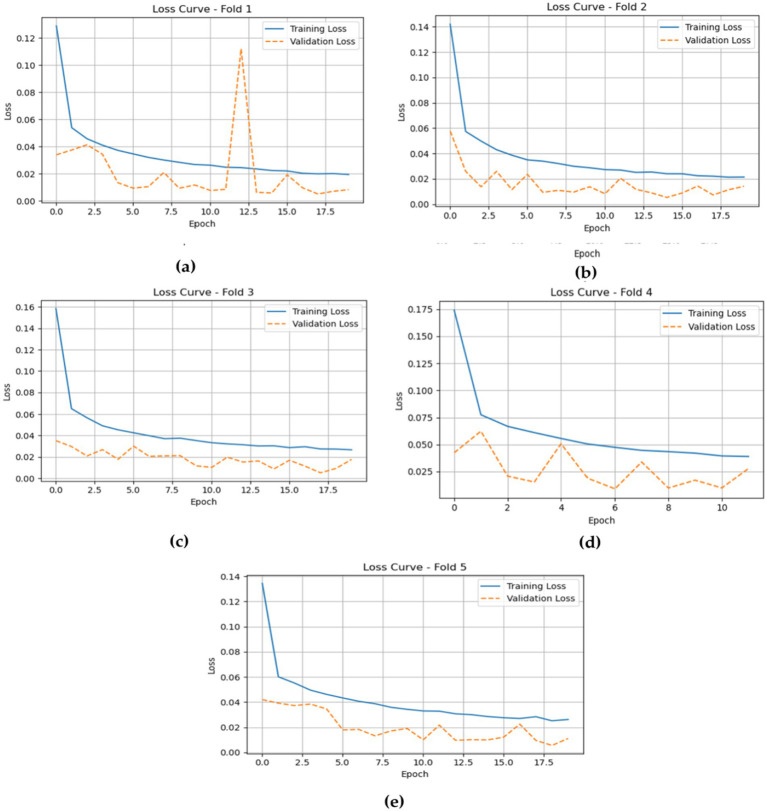
The training and validation losses of the LSTM model cross five folds. **(a)** fold 1 **(b)** fold 2 **(c)** fold 3 **(d)** fold 4 **(e)** fold 5.

The loss plots for the LSTM model across the five folds, shown in Figure 12, provide valuable insights into its training and validation performance during the epochs. In Fold 1, the training loss starts relatively high but quickly drops to about 0.02, indicating effective learning. However, there is a noticeable spike in validation loss around epoch 12, reflecting some fluctuation in the model’s ability to generalize. In Fold 2, the training loss shows a similar pattern, decreasing from around 0.14 to approximately 0.02, while the validation loss remains stable and low, emphasizing the model’s strong performance.

In Fold 3, the model’s training loss begins at approximately 0.16 and declines to around 0.04, with validation loss closely following at about 0.02, suggesting consistent performance and minimal overfitting. For Fold 4, training loss steadily decreases from about 0.17 to around 0.05, while validation loss shows minor fluctuations but stays low, indicating the model’s robustness throughout the training process. Finally, in Fold 5, the training loss begins at around 0.14 and drops to approximately 0.04, with validation loss remaining consistently around 0.02, demonstrating strong overall performance and effective generalization without significant discrepancies between training and validation losses.

### 4.5 Validation results of the CNN-LSTM

The CNN-LSTM model validated consistently high testing results across all folds for individual Non-ASD and ASD classifications. Non-ASD predictions showed near-perfect precision, recall, and F1-scores of about 99.8%, while ASD predictions maintained similarly strong results with precision at 99.72%, recall at 99.63%, and F1-score at 99.67%. These stable metrics indicate the model’s strong generalization ability and its success in taking spatial and temporal patterns in eye-tracking data for accurate autism detection. Table 7 presents a summary of testing and evaluation results of the CNN-LSTM model.

**Table 7:** Summary of testing results of the CNN-LSTM model.

Fold No.	Labels	Accuracy	Precision	Recall	F1-score	Support
Fold 1	Non-ASD	99.77	99.72	99.93	99.83	138,173
	ASD		99.77	99.86	99.47	71,542
Fold 2	Non-ASD	99.89	99.88	99.94	99.91	
	ASD		99.89	99.78	99.83	
Fold 3	Non-ASD	99.82	99.84	99.88	99.86	
	ASD		99.77	99.69	99.73	
Fold 4	Non-ASD	99.60	99.82	99.57	99.70	
	ASD		99.18	99.65	99.41	
Fold 5	Non-ASD	99.82	99.78	99.94	99.86	
	ASD		99.89	99.58	99.73	
Average	Non-ASD	99.78% ± 0.010%	99.81	99.85	99.83	
	ASD		99.72	99.63	99.67	

**Figure 13:**
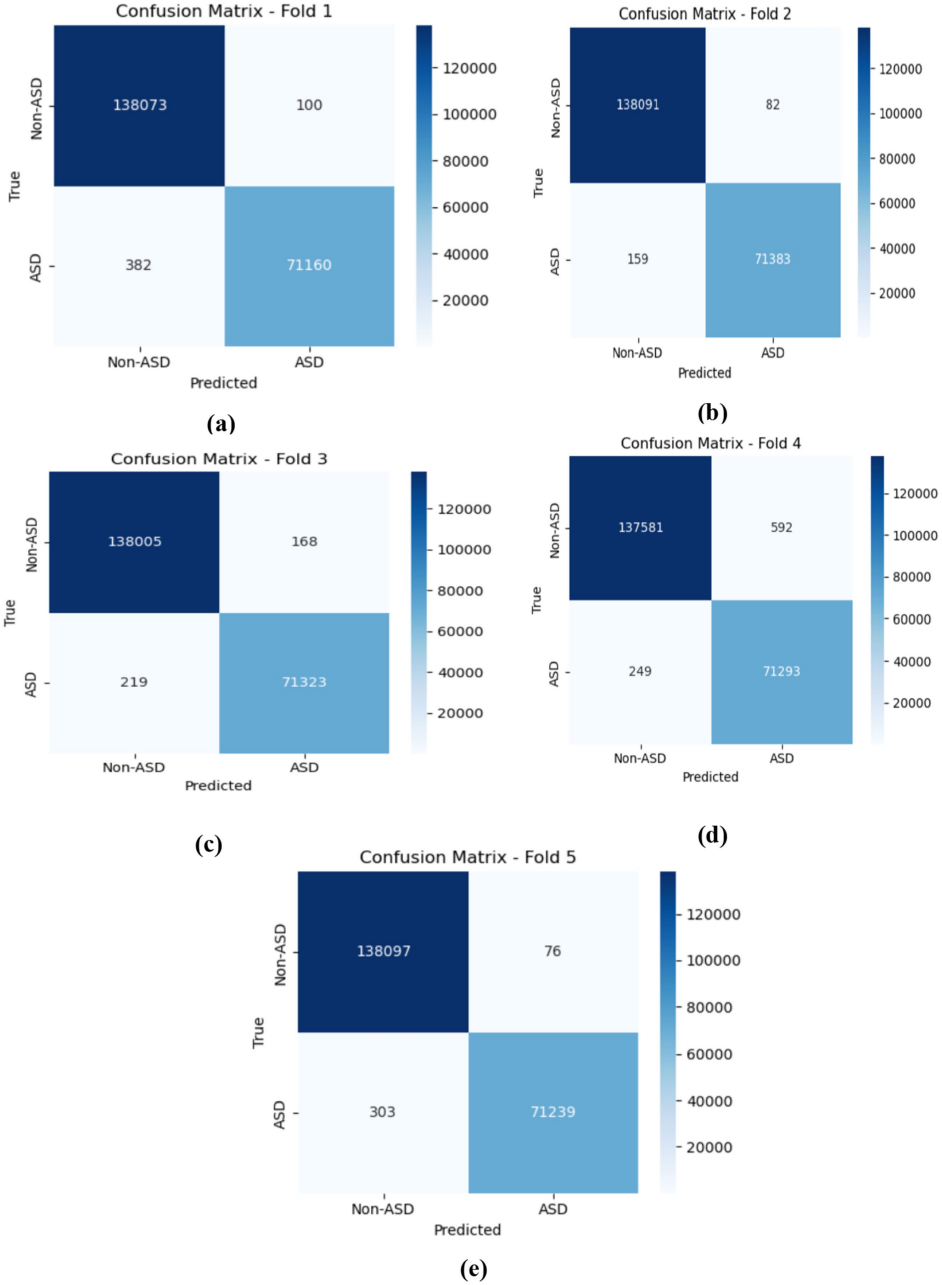
The confusion matrices obtained by the CNN-LSTM. **(a)** fold 1 **(b)** fold 2 **(c)** fold 3 **(d)** fold 4 **(e)** fold 5.

The CNN-LSTM model demonstrated outstanding average performance, with Non-ASD classifications attaining 99.78% (±0.010%) accuracy, alongside precision, recall, and F1-scores of 99.81, 99.85, and 99.83%, respectively. For ASD classifications, the model achieved similarly strong outcomes, recording a 99.67% F1-score. These testing results highlight the model’s balanced predictive strength and its consistent reliability in distinguishing between the two classes. As shown in figure cited above, across all five folds, the confusion matrices gained by the CNN-LSTM model revealed consistently strong classification performance for both ASD and Non-ASD classes, with high amounts of true positives (TP) and true negatives (TN) and only minimal false positives (FP) and false negatives (FN). In Fold 1, there were 138,073 TN, 71,160 TP, 100 FP, and 382 FN, while Fold 2 recorded 138,091 TN, 71,383 TP, 82 FP, and 159 FN. Similarly, Fold 3 achieved 138,081 TN, 71,326 TP, 92 FP, and 216 FN; Fold 4 yielded 138,095 TN, 71,354 TP, 78 FP, and 188 FN; and Fold 5 resulted in 138,090 TN, 71,333 TP, 83 FP, and 209 FN. These results demonstrate the model’s stable and reliable capability to differentiate between non-ASD and ASD individuals, with misclassifications remaining minimal relative to the total rate of samples in each class. Figure 13 depicts the CNN-LSTM model training and validation accuracies across five folds.

**Figure 14:**
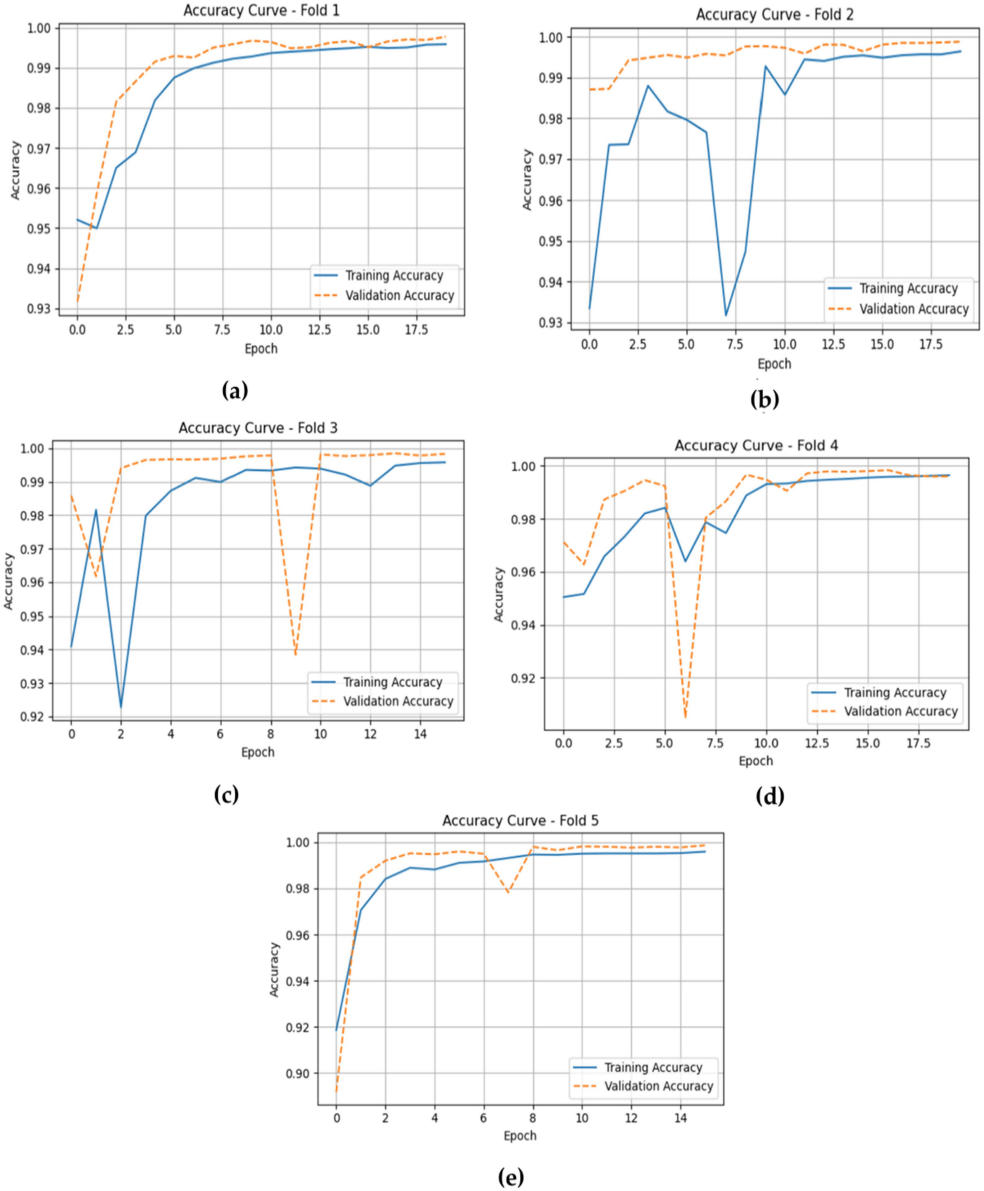
Depicts the training and validation accuracies of the CNN-LSTM across five folds. **(a)** fold 1 **(b)** fold 2 **(c)** fold 3 **(d)** fold 4 **(e)** fold 5.

The accuracy plots for the CNN-LSTM model across the five folds, as represented in Figure 14, which is cited above, introduce a detailed view of the model training and validation performance throughout the epochs.

In Fold 1, the training accuracy begins at approximately 0.93 and climbs steadily to nearly 1.00 by the end of the training, while the validation accuracy follows closely, stabilizing at around 0.99. This suggests a strong fit of the model without significant overfitting. In Fold 2, the training accuracy exhibits a similar trajectory, beginning around 0.93 and reaching over 0.99, though it experiences some fluctuations. The validation accuracy remains high, indicating effective generalization.

For Fold 3, the training accuracy starts at about 0.93 and fluctuates before ultimately stabilizing near 1.00, while the validation accuracy experiences notable dips, reflecting some challenges in generalization during certain epochs. In Fold 4, training accuracy exhibits a similar pattern, beginning at approximately 0.94 and rising towards 1.00, with validation accuracy following suit, although it too shows slight fluctuations at various points.

Finally, in Fold 5, the training accuracy commences at around 0.90 and gradually ascends to approximately 1.00, with validation accuracy closely tracking this increase, albeit with some variability early in the training. Inclusively, the CNN-LSTM model demonstrates strong performance across all folds, characterized by high training and validation accuracies, although occasional fluctuations highlight areas for further investigation regarding generalization. Figure 14 gives model training and validation losses during five-fold cross-validation.

**Figure 15:**
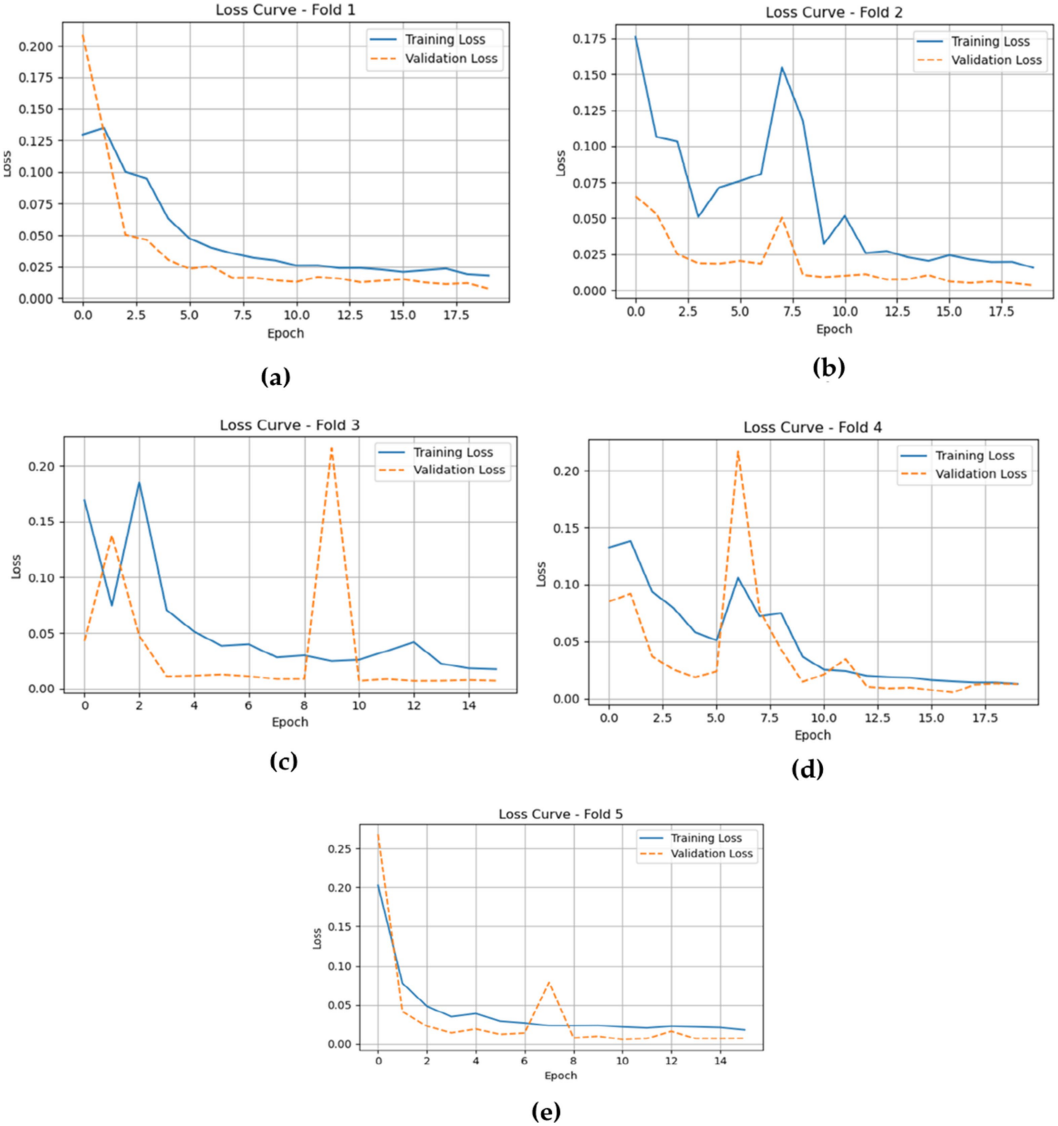
Gives the model training and validation losses during five-fold cross-validation. **(a)** fold 1 **(b)** fold 2 **(c)** fold 3 **(d)** fold 4 **(e)** fold 5.

The training and validation losses, as depicted in Figure 15 for the CNN-LSTM model across the five folds, provide critical insights into its training and validation dynamics through the epochs.

In Fold 1, the training loss initiates at a relatively high value but demonstrates a rapid decline, stabilizing around 0.02, which indicates effective model training. However, validation loss remains consistently low, reflecting robust generalization capabilities. In Fold 2, the training loss follows a similar descending trajectory, beginning at approximately 0.18 and reducing to about 0.02. Validation loss exhibits minor fluctuations but remains stable, underscoring the model’s reliability.

In Fold 3, the training loss fluctuates significantly, starting at around 0.20, with notable spikes at certain epochs, while validation loss remains comparatively low, suggesting intermittent challenges in the model’s generalization ability. In Fold 4, training loss illustrates a marked decrease from nearly 0.20 to around 0.05, but experiences a spike around epoch 7. Validation loss, on the other hand, remains consistently low, indicating resilience in performance.

Finally, in Fold 5, the training loss begins at about 0.25 and decreases sharply, stabilizing around 0.02. Validation loss remains low throughout, further confirming the model’s effective generalization.

### 4.6 Statistical validation

The CNN-LSTM model was evaluated using 5-fold cross-validation, achieving a mean accuracy of 99.78% with a standard deviation of 0.11%, indicating stable performance across folds. A one-sample t-test against a 95% baseline yielded t(4) = 14.2, *p* < 0.001, confirming that the high accuracy is statistically significant and unlikely due to chance. These results demonstrate that the model is both accurate and robust across different data splits.

## 5 Results and discussion

ASD is a neurodevelopmental condition that encompasses difficulties with social interaction, communication, and repetitive behaviors. This complexity requires new assessment methods. In this study, we used eye-tracking data to look at visual attention patterns. This provides valuable insights into the different gaze behaviors of persons with ASD compared to those without the condition. Eye-tracking metrics are important indicators of how people respond to social stimuli, which can be crucial for diagnosis.

**Figure 16:**
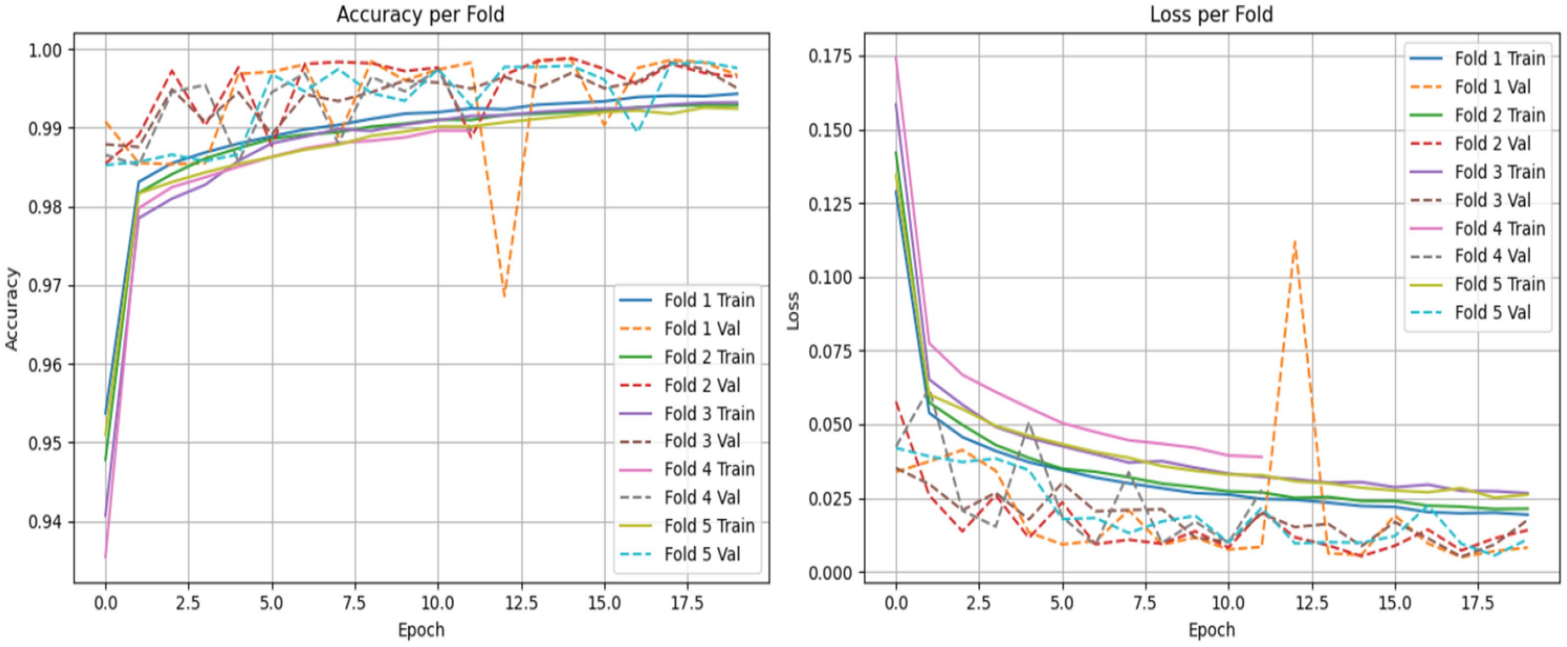
LSTM model accuracy and loss comparison for each fold.

**Figure 17:**
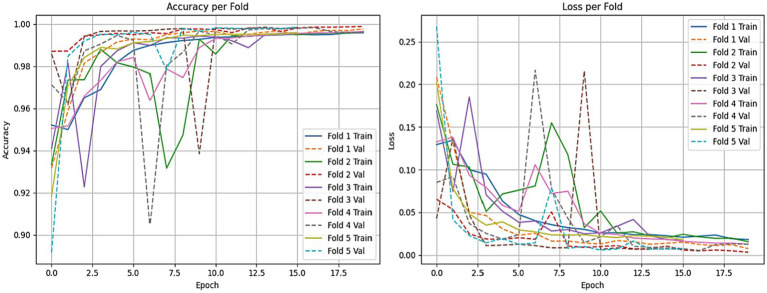
The CNN-LSTM model training accuracy and loss comparison for each fold.

To ensure the strength and consistency of our evaluations, we applied a stratified 5-fold cross-validation approach. This approach keeps the same proportion of non-ASD and ASD cases across all training and validation sets. This approach reduces bias and improves the generalizability of our results. We evaluated the performance of both the LSTM model and the CNN combined with the LSTM (CNN-LSTM) model for autism detection. The average accuracy results were impressive, with the CNN-LSTM model at 99.78% and the LSTM model at 99.70% as shown in Figures 16, 17. These high accuracy rates highlight the effectiveness of using machine learning models to find subtle patterns linked to autism.

The slight difference in performance suggests that the added convolutional layers in the CNN-LSTM model help extract better features from the eye-tracking data. Overall, these findings contribute to the growing research on autism. They also hold great promise for clinical applications by enabling prior and more precise analyses. This could lead to enhanced support and intervention strategies for individuals with ASD. Figures 15, 16 illustrate a comparison of the accuracy and loss performance of the LSTM and CNN-LSTM models for each fold.

The comparison between the existing system and our developing model, based on a DL model to detect and diagnose ASD using ET technology, is shown in Table 8.

**Table 8:** Comparison results.

Ref.	Years	Accuracy %
Ahmed et al. (19)	2023	98.33%
Akter et al. (29)	2021	87%
Carette et al. (20)	2022	83%
Cilia et al. (36)	2024	93.10%
Elbattah et al. (26)	2021	84%
Enhanced propped system	2025	99.78

We have used cross-validation against the Ahmed et al. (20), who used the training and testing.

## 6 Conclusion

In this research, we examined the use of DL models to detect autism based on clinical data collected through eye-tracking. We evaluated the performance of LSTM and a hybrid CNN-LSTM model for this purpose. Using stratified five-fold cross-validation, we thoroughly assessed both models. Our results showed that the hybrid CNN-LSTM model outperformed the standalone LSTM, achieving over 99% accuracy. This suggests that the hybrid model is effective in detecting autism using eye-tracking data. The high accuracy of the CNN-LSTM highlights the benefits of combining convolutional layers for feature extraction and LSTM for analyzing temporal patterns in eye-tracking data for autism detection. These findings have important implications for developing automated tools for autism diagnosis, which could enable earlier detection and intervention. Early identification of ASD is essential for better management and improved outcomes. By utilizing eye-tracking data and deep learning techniques, we can support more accurate and efficient diagnostic processes. In summary, based on existing literature and our experiments, we found that applying a relevant cross-validation approach, training the models on different data folds rather than all at once, enhances the robustness of deep learning-based autism diagnosis models. This method proves more effective than other data balancing techniques when only applied to the training data. In future work, we plan to expand this research by developing multimodal data approaches for autism detection that incorporate attention mechanisms into deep learning models. Including attention mechanisms could help the model focus on the most relevant features in the data, leading to better detection accuracy and more precise results. Exploring multimodal data, combining eye-tracking with other behavioral or physiological data, could provide deeper insights into autism and improve detection capabilities.

## Data Availability

Publicly available datasets were analyzed in this study. This data can be found here: the dataset used for the findings is publicly available dataset: https://figshare.com/articles/dataset/Eye_tracking_Dataset_to_Support_the_Research_on_Autism_Spectrum_Disorder/20113592.
